# Effect of TaC Content on Microstructure and Properties of W-TaC Composites

**DOI:** 10.3390/ma16010186

**Published:** 2022-12-25

**Authors:** Kai Xu, Yaning Zhang, Dong Wang, Xing Jin, Xiang Ding

**Affiliations:** 1 Anhui Province Key Laboratory of Metallurgical Engineering & Resources Recycling, Anhui University of Technology, Maanshan 243002, China; 2 School of Materials Science and Engineering, Anhui University of Technology, Maanshan 243002, China

**Keywords:** W-TaC, Vickers hardness, flexural strength, thermal conductivity, plasma ablation

## Abstract

Transition metal carbide reinforcement can improve the performance of pure W. W-(10–50) vol% TaC composites were prepared by spark plasma sintering at 2100 °C. The effect of TaC content on the microstructure, mechanical properties, and thermal conductivity of the composites was studied. The ablation resistance of the W-TaC composites was evaluated under an air plasma torch. The addition of TaC into the W matrix enhanced the densification of W-TaC composites, the density of W-40 vol% TaC composite exceeded 93%. TaC particles inhibited the growth of W grains during sintering. Reactive diffusion occurred between W and TaC, forming the solid solutions of (W,Ta)_ss_ and (Ta,W)C_ss_. W and TaC react to form the W_2_C phase at a TaC content of 50 vol%. The Vickers hardness of the composite increases from 3.06 GPa for WTA1 to 10.43 GPa for WTA5. The flexural strength reached 528 MPa in the W-40 vol% TaC composite. The thermal conductivity of W-20 vol% TaC composite was 51.2 ± 0.2 W·m^−1^·K^−1^ at 750 °C. The addition of TaC improved the ablation resistance of W-TaC composites. The mass ablation rate of W-30 vol% TaC composite was 0.0678 g·s^−1^. The ablation products were mainly W oxides and complex oxides of W-Ta-O.

## 1. Introduction

With the development of advanced nuclear reactors, aerospace, and other fields, materials for extreme environments (such as plasma facing materials for fusion reactor and thermal protection materials in ultra-high speed aircraft) are quite demanded. Refractory metals and their alloys, carbon–carbon composites, and ultra-high temperature ceramics (carbides, nitrides, and borides of the IVB–VIB groups of transition metals) are common candidates [1,2,3,4,5]. Tungsten (W) has the highest melting point of 3422 °C in refractory metals, and its strength is over 800 MPa, and the thermal conductivity reaches 105 ± 10 W·m^−1^·K^−1^ [6,7]. However, the strength of pure W significantly declines at high temperatures with poor ablation resistance, which is challenging in the extreme service conditions of the components. The addition of refractory carbides (ZrC, NbC, HfC, TiC, etc.) can improve the high-temperature strength and ablation resistance of W. The composites possess high thermal conductivity and thermal shock resistance at ultra-high temperatures [8,9,10,11,12]. The strength of the W-40 vol% ZrC composite prepared by hot pressing exceeded 800 MPa at 1200 °C, and the density was only 2/3 that of pure W. The mass ablation rate of the W-30 vol% HfC composite was 0.0157 g·s^−1^, which was only 1/3 that of pure W [13]. Among the family of refractory carbides, TaC has a melting point as high as 3880 °C and good high-temperature stability [14]. However, W-TaC composites were rarely reported in the literature.

Teague et al. prepared W-49 vol%Ta_2_C composites by in situ reactive sintering, with a micro Vickers hardness of 13.4 GPa, a flexural strength of more than 584 MPa, and a fracture toughness of 8.3 MPa·m^1/2^ [15,16]. Jiang. et al. prepared a W-TaC composite with a density of 84.7%, whose mass and linear ablation rates were 0.0048 g·s^−1^ and 0.0233 mm·s^−1^ [17], respectively. Compared with hot pressing and in situ reactive sintering, spark plasma sintering (SPS) technology has the advantages of a fast heating rate and short sintering time [18]. Miao et al. used SPS to prepare TaC-dispersion-strengthened W-based materials with a TaC volume fraction less than 1% and whose Vickers hardness was over 4.57 GPa, tensile strength exceeded 370 MPa at 600 °C, and thermal conductivity reached 135 W·m^−1^·K^−1^ [19]. The results showed that the TaC particles in the composites could effectively pin the dislocations, hinder the movement of grain boundaries, and therefore improve the strength of the composites. In addition, Ta and C react with oxygen in the matrix to form Ta-C-O compounds, which played a role in grain boundary purification [20,21]. However, there are few reports on SPS-ed W-TaC composites with TaC volume fractions over 10%. The effect of TaC content on the microstructure and properties of W-TaC composites was unclear.

In this research, W-TaC composites with increasing TaC content from 10 vol% to 50 vol% were prepared by SPS, and the effects of TaC content on the microstructure, mechanical properties, thermal conductivity, and ablation resistance were investigated.

## 2. Materials and Methods

Commercial W and TaC powders were used as raw materials with average particle sizes of 1 μm (W) and 0.5 μm (TaC), respectively. The mixed powders of W and TaC were weighed and milled in polyethylene tanks using Si_3_N_4_ balls as the milling medium. The ball-to-powder ratio was 3:1. After milling for 24 h, the mixed powders were passed through a 200 mesh sieve. The mixed powders with designed compositions were then put into the graphite mold and densified into composite discs (30 mm in diameter and 3.5 mm in thickness) using discharge plasma sintering (SPS-10T-10-IV). The sintering was performed at 2100 °C for 10 min with a pressure of 40 MPa. The heating and cooling rates were 100 °C·min^−1^ and 200 °C·min^−1^, respectively. The compositions and codes of the as-prepared composites are listed in Table 1.

The density of the composites was measured by Archimedes’ method, and the porosity was calculated. The phase composition of the specimens was tested using an X-ray diffractometer (XRD, Rigaku UItima IV, Tokyo, Japan) The scanning rate and step were 4°·min^−1^ and 0.02°, respectively. The XRD spectra of the samples were fitted with the ICCD data. The composites were polished to a mirror surface. The microstructure was characterized by scanning electron microscopy (SEM, Tescan, Brno, Czech Republic) equipped with energy-dispersive X-ray spectroscopy (EDS) for elemental analysis. The hardness of the composites was measured using a Vickers hardness tester (load 5 kgf, holding time 10 s, take the average of 10 indentations), and the flexural strength of the composites was tested by the three-point bending method (span 16 mm, loading rate 0.5 mm·min^−1^) on a Shimadzu-type universal testing machine (chamfered specimens with dimensions of 2 mm × 4 mm × 20 mm ). The thermal conductivity of the composites (12.5 mm in diameter and 3 mm in thickness) was tested under flowing argon gas using a laser thermal conductivity tester of type LFA457 with laser voltage of 1538 V and test temperatures of 750 °C, 850 °C, and 950 °C. The ablation resistance of the composites (12.5 mm in diameter and 3 mm in thickness) was estimated by the air plasma torch of an LGK-80YM plasma cutting machine. The distance between the gun tip and the surface of the specimens was 4 mm. The operating power of the air plasma was 8000 W with a current of 50 A and an air pressure of 0.5 MPa. The ablation time was set as 3 s. The mass ablation rate Rm was calculated by the formula: R_m_ = (m_1_ − m_2_) /t, where m_1_ and m_2_ represent the mass of the sample before and after ablation, and t is the ablation time.

## 3. Results

### 3.1. Effect of TaC Content on Phase Composition and Microstructure of W-TaC Composites

**Figure 1:**
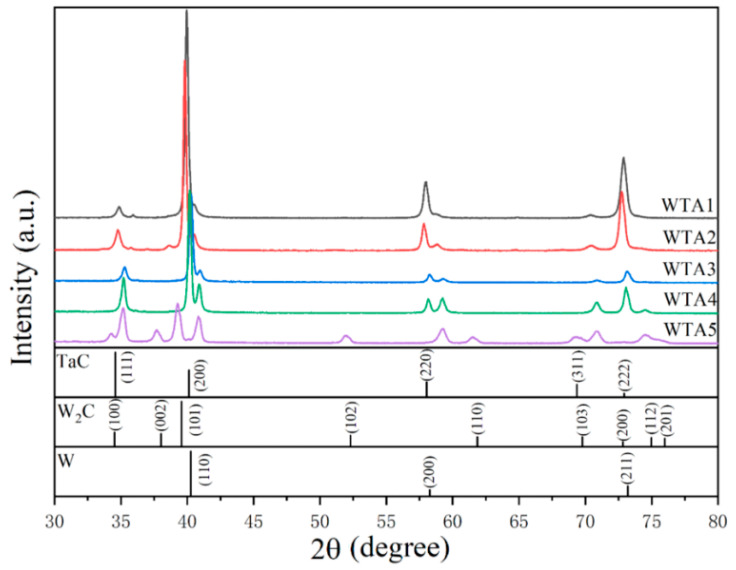
Phase compositions of the as-sintered W-TaC composites.

Figure 1 shows the XRD patterns of the fabricated W-TaC composites. W and TaC are the main phases in all the samples. With the increase of TaC content, the intensity of TaC diffraction peaks gradually increases, and the intensity of the W diffraction peak gradually decreases. Compared with the standard ICDD-PDF cards, the diffraction peaks for W shifted to lower angles. In contrast, the peaks for TaC showed a reverse trend, which indicates the increment in the lattice parameter of W and the reduction in the value of TaC. During high-temperature sintering, Ta (with a radii of 0.143 nm) atoms probably diffused into W (with a radii of 0.137 nm) lattice to form the solid solutions (W, Ta)_ss_ and (Ta,W)C_ss_ [6,12,22]. The diffraction peaks showed a significant change when the TaC content reached 50 vol% in the composite, and the W diffraction peaks declined while the peaks for W_2_C appeared. Combined with the analysis of the W-Ta-C ternary phase diagram, there is a “W+TaC_x_+W_2_C” region in the tie-line between W and TaC when the TaC content is over 10 mol% [23]. Compared with the standard PDF#35-0776, the W_2_C diffraction peak shifted to lower angles, probably because TaC_x_ further interacted with W_2_C to form (W, Ta)_2_C_ss_. The TaC and W_2_C diffraction peaks are shifted by 1°~2°. The SPS non-equilibrium sintering and the lack of C in the TaC and W_2_C cells are also important factors in the diffraction peak shift. It indicates that the W-TaC system has a similar phase compositions to that of the W-NbC system [12].

**Figure 2:**
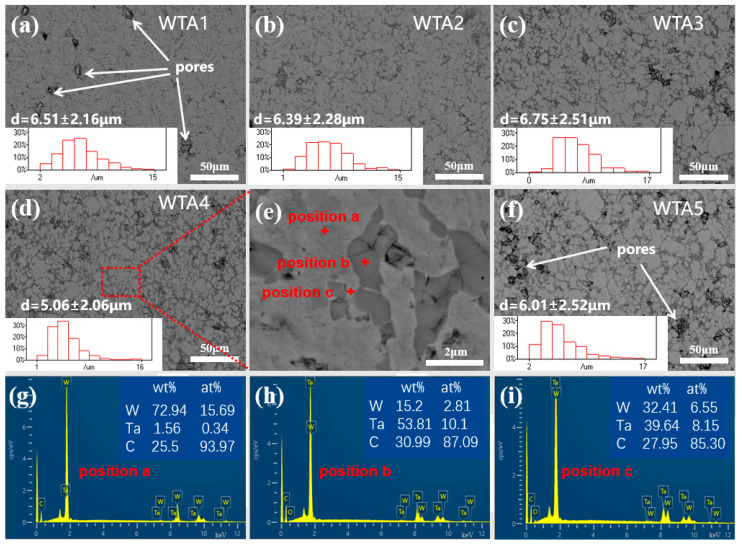
Backscattered electron images of WTA1–WTA5 composites and corresponding EDS results of specific locations in the composite WTA4.

Figure 2a–f shows backscattered electron images taken from the polished surfaces of specimens WTA1–WTA5, respectively. With the increase of TaC content, the relative density increases from 83.2% in WTA1 to 93.7% in WTA4, where the lower densification density in WTA1 and WTA5 is due to the presence of residual pores with uneven sizes, which may be caused by the aggregations of W grains during the sintering process and the difficulty in densification within a relatively short SPS sintering time. In composites WTA2, WTA3, and WTA4, the W grains are relatively uniformly distributed, and the particle size decreases from 6.39 ± 2.28 μm in WTA2 to 5.06 ± 2.06 μm in WTA4. With the increase of TaC content, the refining effect of TaC second-phase particles became obvious by hindering the migration of W grain boundaries and inhibiting the abnormal growth of W grains [6,24]. Combined with EDS results (Figure 2g–i) at specific locations in composite WTA4, the phase in white contrast is W, and the gray phase is TaC. As shown in Figure 2e, TaC particles were sintered to larger ones. However, there are residual pores in the TaC clusters, which could lead to reduced strength. In some large TaC particles, rod-like phases in white contrast were observed. According to the studies in the W-NbC composites [12], such a microstructure was probably caused by the decomposition of the (Ta,W)C_ss_ phase.

### 3.2. Effect of TaC Content on Mechanical Properties

Table 2 collects the mechanical properties of the W-TaC composites. With the increase of TaC content, the density of the composites increases from 83.2% in WTA1 to 93.7% in WTA4. This indicated that the addition of TaC second phase enhanced the densification of the W-TaC composite. Due to the reaction of TaC with W to form W_2_C, the WTA5 composites have a density of 91.6 ± 0.2%, which is similar to those of the W-TiC and W-ZrC composites [6]. The Vickers hardness of the composite increases from 3.06 ± 0.05 GPa in WTA1 to 10.43 ± 0.73 GPa in WTA5, respectively. The hardness of TaC and W are 21 GPa and 3 GPa, respectively [14,25]. The experimental results were consistent with the calculation of the rule of mixture, and the minus deviations were due to unfavorable factors such as porosity and third phases.

With the increased TaC content, the flexural strength of the composites increased from 292 ± 14 MPa for WTA1 to 528 ± 22 MPa for WTA4. The TaC second phase particles effectively increased the strength of the W matrix. The inter-diffusions at the W/TaC interfaces generated (W, Ta)_ss_ and (W, Ta)C_ss_ phases. The strong bonding of solid solution phases between the W and TaC grains positively affects the composite’s strength. Meanwhile, the strength of the W matrix is extensively enhanced by refining and solid solution strengthening. However, excessive TaC reacts with W to form W_2_C, and the TaC particles agglomerate to increase the porosity of the composites, and the poor interfacial bonding reduces the flexural strength of the composites [26].

**Figure 3:**
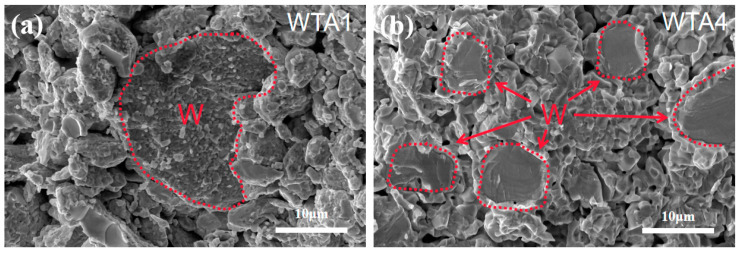
Fracture surfaces of (**a**) WTA1 and (**b**) WTA4.

Figure 3a,b show the fracture surfaces of the composites WTA1 and WTA4. The sub-micron-sized TaC particles are distributed around the W grains with sizes of microns. Compared with WTA1, there are fewer pores, and the size of W grains is reduced, which agrees with the relative density and microstructure. The W phase in composites exhibits a dominant transgranular fracture with river patterns. The TaC rims around the fractured W grains imply strong bondings between the W/TaC interface. The fracture of the TaC phase showed a combination of intergranular and transgranular modes. Some pull-outs of TaC particles were observed. The grain refining effect on W, strong TaC/W interfaces, and bondings between TaC particles has made the strength of WTA4 nearly twice that of WTA1.

### 3.3. Effect of TaC Content on Thermal Conductivity

**Figure 4:**
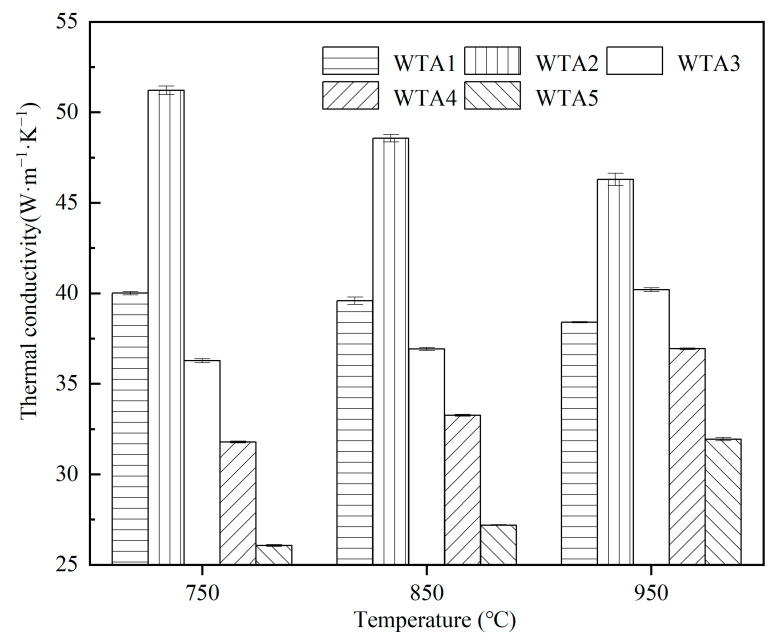
Thermal conductivity of WTA1–WTA5 composites at 750–950 °C.

Figure 4 shows the thermal conductivity of W-TaC composites at 750 °C, 850 °C, and 950 °C. The thermal conductivity increases and then decreases with the rise in TaC content, and WTA2 shows excellent thermal conductivity of 51.2 ± 0.2 W·m^−1^·K^−1^ at 750 °C. The thermal conductivities of W and TaC are ~175 W·m^−1^·K^−1^ and 15–35 W·m^−1^·K^−1^, respectively [14,25]. Consequently, composites WTA1 and WTA2 with more W content have higher thermal conductivities than other samples. WTA2 exhibits higher values than WTA1 due to fewer pores in the scattering of phonons [27]. For WTA3, WTA4, and WTA5, the thermal conductivity of the composites gradually decreases with the increase in TaC content, and the value for WTA5 is lowered to 25.5 ± 0.1 W·m^−1^·K^−1^. This might result from the higher TaC content, distributed in the composite matrix as a second phase, and hinders the thermal conduction between tungsten crystals. Besides, the effect of residual pores on the thermal conductivity of the composite and the existence W_2_C phase with low thermal conductivity (~30 W·m^−1^·K^−1^) is non-negligible [28].

The thermal conductivities of WTA1 and WTA2 decrease with increasing temperature, while the values of WTA3, WTA4, and WTA5 increase. Since the thermal conductivity of W decreases with increasing temperature and those of TaC and W_2_C have opposite trends, the different content of TaC makes the thermal conductivity of W-TaC composites change in different trends at the test temperatures. The increase in temperature increases the chance of collision between free electrons and metal ions, and the thermal conductivity shows a decreasing trend. The higher the W content in WTA1 and WTA2, the stronger decreasing trend of the thermal conductivity of the composites as the test temperature decreases. For WTA3, WTA4, and WTA5 composites with higher TaC contents, the thermal conductivity turns to an increasing trend with temperature. In WTA5, W reacts with TaC to form W_2_C, and the thermal conductivity is increased more significantly than those of WTA3 and WTA4.

### 3.4. Effect of TaC Content on Anti-Ablation Properties

**Figure 5:**
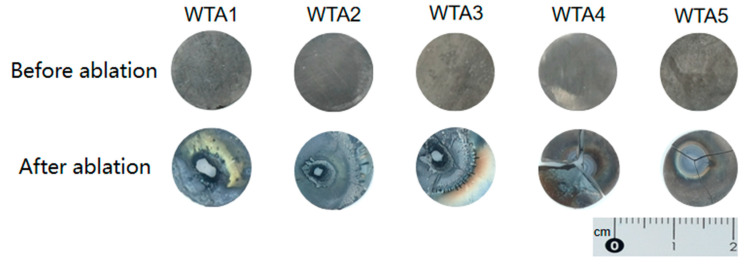
Macroscopic topography of WTA1–WTA5 composites before and after ablation.

Figure 5 shows the macroscopic morphology of the W-TaC samples before and after air plasma ablation. The composites WTA1, WTA2, and WTA3 withstood and were maintained after sharp heating and following ~14k °C [29] ultrahigh-temperature ablation for 3 s, while samples with TaC contents over 40 vol% fragmented to pieces, implying reduced thermal shock resistance with higher TaC contents. A light yellow smoke accompanied the ablation process, and there were prominent light yellow products around the ablation pits of the samples (obviously seen in WTA1). The melting point and boiling point of tungsten oxides are relatively low (e.g., 1852 °C for WO_2_) [30]. As a result, the evaporation of tungsten oxides occurred during the ablation process. The droplet-like splash products around the ablation pits are due to the molten oxides of W and TaC in the composites being blown by the arc gas flow that re-solidified in regions adjacent to the ablation center [6,17]. Compared with the composites with less TaC content, there is hardly any sputtering out of the ablation products on the surfaces of composites WTA4 and WTA5. Hasselman proposed the thermal shock resistance parameter Rst to evaluate the ability of a composite to resist crack propagation [31]:

R_st_= (G/α^2^E)^1/2^

where G, α, and E are the work of a fracture over a large area, the thermal expansion coefficient, and the elastic modulus of a material, respectively. In WTA4 and WTA5, the higher TaC content and the W_2_C phase enhance the composite’s thermal expansion coefficient. It is difficult to get rid of the more considerable thermal stresses during quick heating at the beginning of the ablation process [32].

The mass ablation rates of W-TaC composites were collected in Table 3. The addition of TaC has improved the ablation resistance of the W-TaC composites. The tested value reduced from 0.0918 g·s^−1^ in WTA1 to 0.0678 g·s^−1^ in WTA3. In the composite WTA4, it declined to a negative value of −0.0126 g·s^−1^. The negative values are mainly due to the thermal shock damage of WTA4 and WTA5 at the moment of ablation, but the ablation was not continued. In the very short time from the beginning of ablation to the thermal shock damage, a small amount of microstructure in the ablation center is oxidized, which leads to the increase in the mass of the composites after ablation. The ablation rate was higher than that (0.0048 g·s^−1^) of the reactive sintered W-30 vol%TaC composite ablated under an oxyacetylene flame [17]. The different testing conditions are probably the main reason.

**Figure 6:**
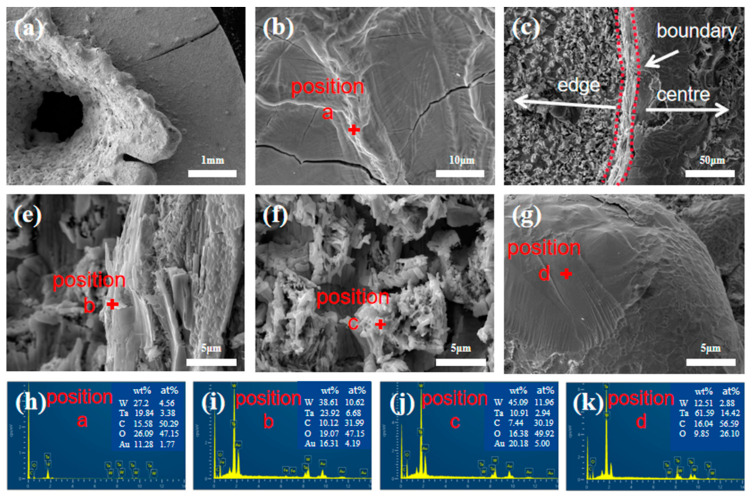
Microstructure and EDS results at corresponding locations in the WTA3 composite after air plasma ablation.

Figure 6 shows the microstructure and EDS results of the WTA3 composite after air plasma ablation. There was a round table shape with uneven droplet-like sputtered products around the central ablation crater (seen in Figure 6a). There is a micro-crack in the upper right corner. This could be due to the enormous internal thermal stress. The ablation products covering the edge of the ablation crater were dense (seen in Figure 6b), which was analyzed with the EDS as a mixed oxide of W and Ta, possibly including WO_3_, Ta_2_WO_8_, etc. [17] There is a demarcation area between the center region and the edge of the ablation surface, as shown in Figure 6c. The ablation products in areas near the center are mainly lamellar oxides. At the same time, those near the edge are very different from that of the boundary area, especially oxides of W, as indicated by the EDS analysis in Figure 6e,f. Figure 6g shows the teardrop-like organization sputtered during the ablation process, mainly the oxide of Ta by the EDS analysis. From the EDS results, it is clear that the oxide content of W gradually increases from the ablation center to the edge region, probably since oxides of W are more volatilized than oxides of Ta (the melting point of Ta_2_O_5_ is 400 °C higher than that of WO_3_) [30]. Oxides containing more Ta could be sputtered out by the arc flow during the ablation process, which formed droplets in lower-temperature regions around the ablation center.

## 4. Conclusions

Composites were prepared by SPS sintering with TaC as the second reinforcing phase. The results show that TaC improves the mechanical and thermal properties of W-based materials, and the main conclusions are as follows:

1. The densities of the composites exceeded 93% when the TaC content was 40 vol%, and W reacts with TaC to form (W, Ta)C_ss_ + W_2_C + (W, Ta)_2_C_ss_ due to solid solution reaction, TaC and W_2_C cell C deficiency, and non-equilibrium sintering when the TaC content was 50 vol%.

2. The hardness of the composites increased with the increase in the TaC volume fraction, and when the TaC content was 50 vol%, the hardness of the composites reached 10.43 ± 0.73 GPa. The flexural strength of the W-40 vol%TaC composite reached a maximum of 528 ± 22 MPa.

3. The thermal conductivity of the composites increased and then decreased with the increase in the TaC volume fraction. The thermal conductivity of W-20 vol% TaC at 750 °C was 51.2 ± 0.2 W·m^−1^·K^−1^, which decreased with increasing temperature.

4. The addition of TaC enhanced the ablation resistance of W-TaC composites. The mass ablation rate reduced from 0.0918 g·s^−1^ to 0.0678 g·s^−1^ with increasing TaC content from 10 vol% to 30 vol%. The ablation products were mainly W oxides and mixed oxides of W and Ta.

## Figures and Tables

**Table 1:** Materials design and the codes of the composites.

TaC Fraction (vol%)	10	20	30	40	50
Material code	WTA1	WTA2	WTA3	WTA4	WTA5

**Table 2:** Mechanical properties of W-TaC composites.

Composite	Relative Density (%)	Rule of Mixture	Hardness (GPa)	Flexural Strength (MPa)
WTA1	83.2 ± 0.1	4.8	3.06 ± 0.05	292 ± 14
WTA2	86.9 ± 0.3	6.5	4.18 ± 0.03	304 ± 20
WTA3	89.6 ± 0.2	8.4	4.93 ± 0.18	396 ± 5
WTA4	93.7 ± 0.1	10.2	7.75 ± 0.08	528 ± 22
WTA5	91.6 ± 0.2	12.0	10.43 ± 0.73	449 ± 2

**Table 3:** Mass ablation rates of the W-TaC composites.

Composite	WTA1	WTA2	WTA3	WTA4	WTA5
Mass ablation rate (g·s^−1^)	0.0918	0.0569	0.0678	−0.0126	−0.0007

## Data Availability

Not applicable.
